# Comprehensive support for families with parental cancer (Family-SCOUT), evaluation of a complex intervention: study protocol for a non-randomized controlled trial

**DOI:** 10.1186/s13063-021-05577-y

**Published:** 2021-09-15

**Authors:** Marc Dohmen, Andrea Petermann-Meyer, Daniel Blei, Rebecca Bremen, Evamarie Brock-Midding, Manuela Brüne, Franziska Geiser, Burkhard Haastert, Sarah Maria Halbach, Christian Heuser, Steffen Holsteg, Lina Heier, Andrea Icks, Andre Karger, Joseph Montalbo, Hannah Nakata, Jens Panse, Till-Philip Rottmann, Kristina Sättler, Anja Viehmann, Markus Vomhof, Nicole Ernstmann, Tim H. Brümmendorf

**Affiliations:** 1 Department of Hematology, Oncology, Hemostaseology and Stem Cell Transplantation, Faculty of Medicine, RWTH Aachen University, Aachen, Germany; 2 Center for Integrated Oncology, Aachen Bonn Cologne Duesseldorf (CIO ABCD), Germany; 3 Department of Psychosomatic Medicine and Psychotherapy, University Hospital Bonn, Bonn, Germany; 4 Center for Health Communication and Health Services Research, Department of Psychosomatic Medicine and Psychotherapy, University Hospital Bonn, Bonn, Germany; 5 Institute for Health Services Research and Health Economics, Center for Health and Society, Faculty of Medicine, Heinrich Heine University Düsseldorf, Duesseldorf, Germany; 6 mediStatistica, Neuenrade, Germany; 7 Clinical Institute of Psychosomatic Medicine and Psychotherapy, Faculty of Medicine, University Hospital Düsseldorf, Heinrich Heine University Düsseldorf, Duesseldorf, Germany; 8 Institute for Patient Safety (IfPS), University Hospital Bonn, Bonn, Germany

**Keywords:** F-SCOUT, Family intervention, Minor children, Cancer, Parents, Parental cancer, COSIP (Children of Somatically Ill Parents), CIO^ABCD^, Study protocol, Intervention study

## Abstract

**Background:**

Families with minor children affected by parental cancer are at risk of considerable emotional and organizational stress that can severely burden all family members. So far, there has been a lack of comprehensive support services for affected families. The aim of this project is to implement and evaluate a complex psychosocial intervention for these families by providing advice, information, and care on an emotional, psycho-social, and communicative level during and after the cancer experience and across healthcare sectors.

**Methods:**

Family-SCOUT is a project supported by the German Innovation Fund (https://innovationsfonds.g-ba.de/). The evaluation is based on a mixed-methods quasi-experimental design with the intervention and control groups. A standardized postal survey at three measurement points (T0: study enrollment; T1: 3 months of follow-up; T2: 9 months of follow-up), secondary data from the participating health insurance funds, and semi-structured qualitative interviews are used for summative and formative evaluation. The study aim is to include *n*=560 families. Data will be analyzed according to the intention-to-treat principle. The primary analysis is the comparison of the Hospital Anxiety and Depression Scale (HADS) response rates (minimal important difference (MID) ≥ 1.6 in at least one of the two parents) at T2 between the intervention and control group using Fisher’s exact test. The conduct of the study as well as the development and implementation of the intervention will be accompanied by comprehensive study monitoring following the principles of an effectiveness-implementation hybrid study.

**Discussion:**

The results will allow to test the effectiveness and efficiency of the intervention for the target group. The first experience with the implementation of the intervention in model regions will be available. The evaluation results will serve as the basis to assess the need of including the intervention in the catalog of services of the statutory health insurance funds in Germany.

**Trial registration:**

ClinicalTrials.gov, NCT04186923. Retrospectively registered on 4 December 2019.

**Supplementary Information:**

The online version contains supplementary material available at 10.1186/s13063-021-05577-y.

## Background

Each year, approximately 100,000 children (50,000–150,000) in Germany experience that one of their parents develops cancer (expert estimate, Robert Koch Institute, 2017). In a representative survey in the USA, 14% of cancer patients had underage children [1]. The sudden interruption of familiar everyday routines and the noticeable sense of uncertainty are expected to double the risk for the development of psychological symptoms (anxiety, depression, and psychosomatic complaints) in children confronted with parental cancer compared to children in the general population [2]. These symptoms tend to be rare at the time of diagnosis and often develop during the course of the parent’s cancer treatment or follow-up [3]. In addition, unaffected parents providing support also show remarkably high-stress levels [4] along with a significantly increased risk of morbidity [5]. In a Danish study, a significant increase in in-patient hospital stays due to affective disorders in partners of breast cancer patients was demonstrated over a 13-year follow-up period [6].

To date, the standard support provided in Germany covers domestic help for a maximum of 26 weeks, when children under the age of 12 live in the household. However, the sick leave times of severely ill parents are often much longer. Furthermore, as this type of support is directly linked to the patients’ health insurance status, this sole orientation towards the parent suffering from cancer himself results in the paradox that, at the moment of the patients’ death, support will end immediately, which is the exactly the point in time when remaining family members are at the highest need for support. Consequently, the burden on the other family members and their increased risk for the development of subsequent and potential long-term sequelae and even more so, the initiation of preventive measures thereof are not adequately addressed. Although numerous studies have defined stress factors, risk patterns, and effective interventions for families with a severely ill parent [7], they are neither integrated into standard care nor into prevention plans in Germany. The problem can be further accelerated by the fact that support offered by cancer counseling centers is rarely used by the often-overburdened families, while special services for children are only provided within selected cancer counseling centers [8]. Along this line, hospitals’ social service staff is often not adequately equipped for complex interventions, and potentially helpful interventions offered during or close to the time of initial diagnoses are often neglected [2]. Additionally, access to support by social workers outside of hospital care is hard to find leading a substantial “cross-sectional” gap in the availability of psychosocial care. This phenomenon is somewhat further complicated by the current shift towards more outpatient systemic cancer treatments.

The limited links between the different sectors and the complexity of different service providers involved ultimately lead to structurally insufficient care for the families. Parents are often under severe emotional and organizational stress and therefore often fail to organize the support needed for themselves. Consequently, during the subsequent course of the disease, they may acquire increased morbidity and mortality risks [6, 7]. Risk factors for increased psychological impairment are dysfunctionality in the family and the psychological burden on the parents. In particular, the ease up on psychological burdens for parents, the availability of possible coping strategies, functioning family structures, protective relationships, and open communication within the families are more decisive for the later outcome for all surviving members of the family than medical factors such as prognosis and severity of the disease [9, 10]. This is the starting point for the cross-sectional, multi-professional initiative “family-SCOUT”.

### Description of the intervention

#### Intervention: Family-SCOUT

In order to describe family-SCOUT, we used the TIDieR-checklist [11] for complex psychosocial interventions. The main goal of family-SCOUT is to provide support for families with minors suffering from parental cancer. Both parents (or a single affected parent) should have the chance to reduce stress levels while the overwhelming burden and parentification of their children should be avoided. Parents’ capabilities to emotionally support their children in coping with the new situation should be strengthened. Furthermore, resources allowing the parents themselves to establish the adaptations of their daily life required should be provided. Mental disorders particularly also in the later life of all family members should be prevented.

#### Active outreach support

Patients with an oncological disease and underage children will be identified by physicians, psycho-oncologists, or social workers working in the field of medical, i.e., oncological care. If consented by the affected patient, a member of the family-SCOUT-staff will be contacted. Subsequently, families will be offered an initial counseling session either within the hospital, at the outpatient clinic of practice, or at the families’ home in order to allow organizationally and emotionally overburdened families to make use of available support structures and interventions.

#### Permanent contact person/family-SCOUT

The family-SCOUT provides a continuous contact throughout the entire course of disease including palliative situations, dying, death, and bereavement depending on the family’s need. Professionals working as family-SCOUTs have different backgrounds such as social workers or nurses with additional qualifications in psychotherapy. Additional training involves communicative skills, training in psycho-oncology, developmental psychology, and social law (80 h). The main tasks of the family-SCOUTs are related to all topics covering the family’s concerns, e.g., difficulties in organizing child care, needs for household support, self-assessment of communication behavior in the family, and ways of coping. The family-SCOUTs provide information about all options available for organizational, emotional, and communicative purposes and facilitate access to existing support services, e.g., domestic help, youth welfare services, cancer counseling services, and psycho-oncological services either face-to-face or by telephone. The family-SCOUTs also foster open discussions about the disease between parents and subsequently all family members and provide appropriate brochures and recommendations for reading materials designed for children.

Periods of intervention depend on the affected family’s needs; the observation time within the study is at least 9 months. In palliative situations and in the case of death, companionship by the family-SCOUT continues until stable everyday structures have become re-established and necessary support (e.g., psychotherapeutic support) can be securely offered within a stable framework. If single parents with an unfavorable prognosis are in need, support is provided for arranging a custody declaration in the case of death.

Through the outreach program of the Center for Integrated Oncology (CIO) Aachen-Bonn-Cologne-Duesseldorf (CIO^ABCD^), the family-SCOUT initiative will be closely connected regionally with oncologists in the inpatient and outpatient sectors, social services in the local hospitals, psycho-oncologists, youth welfare staff, pediatric psychotherapists, pediatric psychiatrists, and medical/psychological psychotherapists with training in family therapy.

#### Specific family-centered and child-centered therapeutic interventions

When reaching their limits in open discussions with the families, the family-SCOUTs may involve specially trained therapists working on the basis of the COSIP (Children of Somatically Ill Parents) manual [12]. They will reinforce the parents’ skills, promote open discussion within the family, and support the children in coping with the given scenario by taking development-psychological aspects into account in an age-appropriate fashion. The setting may involve parental sessions, family sessions, and children sessions. No prior ICD diagnosis is required to receive the sessions fostering the preventive approach of the family-SCOUT concept.

#### Family-independent tasks of the family SCOUT

In order to provide support, the family-SCOUTs engage in networking with the staff of medical or social sectors and managing interfaces between the in-patient and outpatient sectors, psycho-oncologists, pediatric psychotherapists, pediatric psychiatrists, and between families and public services (school, kindergarten) as well as the healthcare system and the youth welfare system.

## Methods/design

### Trial design

Through a pilot project in Aachen, this new form of care described here has already completed the phases of theory formation, modeling, and exploratory testing, including phase II in accordance with Campbell et al. [13] and feasibility testing or piloting in accordance with MRC (Medical Research Council) [14] under the name “Brückenschlag.” Within the follow-up project family-SCOUT, interventional levels described are now to be modeled in an appropriately complex evaluation design in order to test the benefits and appropriateness [15] and the degree of success of the intervention’s implementation. This study design combines elements of clinical effectiveness and implementation research in an effectiveness-implementation hybrid study type 2 [16]. The family-SCOUT effectiveness evaluation is based on a quasi-experimental, non-randomized control group design with two study arms (an intervention group (IG) and a control group (CG)), as well as one time point for preliminary measurement and two time points for follow-up measurements.

### Participants

**Fig. 1:**
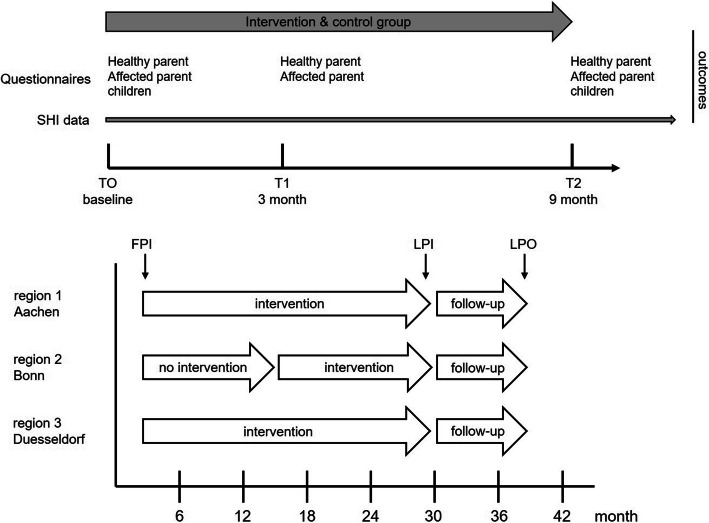
Flowchart of participant flow through the trial and allocation of the intervention to the recruitment sites. SHI statutory health insurance, FPI first patient in, LPI last patient in, LPO last patient out

The study participants will be recruited consecutively in four regions over the recruitment period. Since the support structure in Aachen already existed due to the pilot project, it was ethically not reasonable to withhold the existing service from the patients there; thus, the other regions serve as the control group. In order to be able to depict the implementation of the intervention in another region, the allocation in Bonn was changed during the project. The control group was recruited in the first project phase (during the development of the intervention infrastructure) and the intervention group in the second phase (when the new supporting structures were available). Accordingly, participants in the control group had the option to switch to the intervention group later. In Aachen, the families will be recruited through the Center for Integrated Oncology (CIO) Aachen (CIO^A^, region 1). In Bonn, the families will be recruited through the CIO^B^ and through the Bonn Tumor Center (region 2). In Düsseldorf, the families will be recruited through CIO^D^, region 3) (Fig. 1). In addition, families will be recruited for semi-structured interviews in the Bad Oeynhausen region through the Specialist Clinic for Oncological Rehabilitation and Follow-up Rehabilitation in Bad Oexen (region 4).

#### Inclusion criteria

Confirmed ICD diagnosis of cancer in one parent

Custody of at least one underage child (and/or living in the household)

Adequate German language skills

Membership of the affected parent in a statutory health insurance

Informed consent for study participation from the patient and/or healthy parent including linkage to questionnaire data and routine data

#### Exclusion criteria

Withdrawal of consent by the affected or healthy parent

Relevant cognitive limitation, advanced dementia

Membership of the affected parent in a private health insurance

Individuals who are in relationships of dependence or employment to the project managers or their representatives

### Primary and secondary outcomes

**Fig. 2:**
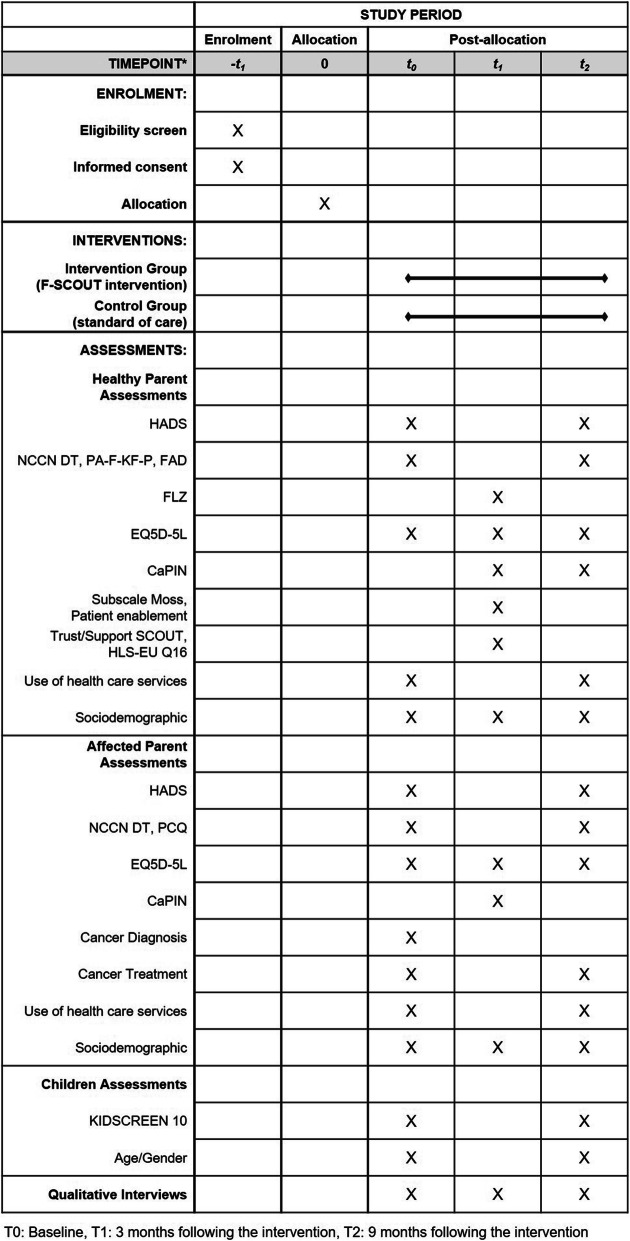
Standard Protocol Items: Recommendations for Interventional Trails (SPIRIT) table of enrolment, intervention, and assessments

The intervention effectiveness will be examined using primary and secondary target variables collected at three measurement points, presented in the variables plan (Fig. 2; see the trial Standard Protocol Items: Recommendations for Interventional Trials (SPIRIT) Checklist for details (in Additional file 1)). The primary endpoint is a reduction in anxiety and depressive symptoms in at least one parent, assessed via the HADS response. HADS response is defined as an HADS reduction between T0 and T2 of at least the minimal important difference (MID) of 1.6.

#### Standardized questionnaires

Participants complete quantitative, self-reported questionnaires including generic data in order to determine the primary and secondary outcome measurements.

#### Statutory health insurance data

Statutory health insurance (SHI) data will be analyzed for all study participants who provided consent and are insured by one of the participating insurance providers. The data include master data, diagnostic data, and treatment data (type of treatment, with the date and costs) of outpatient care by physicians, in-patient care in hospitals, drugs and medication, rehabilitation, reintegration, domestic help, inability to work, domestic nursing care, and nursing services. SHI data covers the time period 12 months before intervention to the complete intervention time. Survey data will be linked with SHI data at the individual level for each respondent who has given consent for data linkage.

#### Semi-structured interviews

Qualitative cross-sectional and sequential interviews with the healthy parent will be carried out in the IG and CG. The semi-structured interview takes approximately 30 min, with a narrative character. Open questions will give the interviewee sufficient scope for the answers. The content will focus on the current family situation, the way the family copes with it, the unmet support needs and available support, and communication aspects within the family. A particular focus in the intervention families will aim at the perceived support provided by the family-SCOUTs. The interviews will be audiotaped, transcribed according to Fuß and Karbach [17], and pseudonymized. The transcripts will be analyzed using Mayring’s qualitative content analysis [18].

## Sample size

On the basis of the project’s pilot phase, it is assumed that 265 families can be included in region 1 (Aachen) in the intervention group (IG). In region 2 (Bonn), it is assumed that a total of 135 families can be included, with 70 families in the control group (CG) in the first year, followed by 65 families in the IG. In region 3 (Düsseldorf), an estimated total of 160 families can be included in the CG. This results in the following sample sizes: IG = 265 + 65 = 330; CG = 70 + 160 = 230. In total *N*=560 families should be included.

A HADS responder rate of around 30% between T0 and T2 is expected in the IG. With the planned sample size, a difference of 28% (IG) versus 16% (CG) will then be identifiable at the 5% significance level with a power of 90% in the full analysis population (after imputation for missing values for dropouts, 330 in the IG, 230 in the CG) using Fisher’s exact test. After exclusion of dropouts of 20% per group between T0 and T1 and again 20% between T1 and T2, sample sizes of 211 (IG) and 147 (CG) remain, such that a complete case analysis could detect a difference from 30 to 16% still with a power of 85%. For the semi-structured interviews, approximately 30–40 healthy partners will be recruited in regions 1–4.

## Statistical methods

Treatment groups will be described by their baseline variables. To investigate potential bias from non-randomization statistical tests corresponding to their distribution (Fisher’s test, *t* test, Wilcoxon test) will be performed to compare both groups. Baseline variables with significant differences will be considered as potential confounders in the following multiple regression analyses.

The primary analysis will be performed using the intention-to-treat principle being a conservative approach in superiority studies. Families enrolled in the CG in Bonn that claims great need for support will be analyzed as belonging to the CG. Only after the expiration of the study period, corresponding to approximately 12 months after inclusion (considering completion of T2 plus corresponding reminders), families may receive the intervention. The observation units will be the families, the parents, or the children. Cluster adjustment for families will be done, if appropriate. The primary analysis is the comparison of HADS response rates on the family level (MID ≥ 1.6 in at least one of the two parents) after 9 months between the IG and CG, using Fisher’s exact test. To adjust for potential confounders in this non-randomized trial, model-based secondary multiple regression analyses will be carried out for the following target variables on family level: HADS response in at least one parent, continuous HADS subscores for depression and anxiety separately for the healthy parent and parent with the disease, DT dichotomized for the cut-off point ≥ 5 for the healthy parent, PA-F-P-KF for the healthy partner, PCQ for the parent with the disease, FAD overall score and subscores for the family, and on children’s level KIDSCREEN for the children aged 8 and upwards. The sociodemographic and disease-specific variables will be included as confounders. Cluster adjustment according to the regions will take place using a corresponding random effect. Supplementary region-specific analyses will be carried out. Differences between the regions will be investigated using an extended interaction model with the independent variables of the region and interaction of the region with IG/CG. In the analysis of the KIDSCREEN questionnaire, the children (≥ 8 years old) will be the observation units, the adjustment will be made for the family cluster using additional random effects. The primary analyses will be carried out after the imputation of missing values of HADS at T0 and T2 by multiple imputations.

The subpopulation with statutory health insurance (SHI) data will be investigated separately. The distribution of baseline variables will be compared between the subpopulations with and without SHI data. The target variables based on the SHI data will be analyzed descriptively and compared between IG and CG on individual level clusters adjusting for families. Costs and uptake will be analyzed in the health economic evaluation. Missing values for the clinical outcomes may arise due to dropouts (not filling the questionnaires at T0–T2, approx. 20% per group) or incomplete details.

### Health-economic evaluation

A health economic evaluation in form of a cost-effectiveness analysis (CEA) and a cost-utility analysis (CUA) will be conducted resulting in an incremental cost-effectiveness ratio (ICER) and an incremental cost-utility ratio (ICUR), respectively. The effect parameter employed in the CEA is the primary outcome of the trial. Thus, the ICER will inform about the additional costs of an additional family with at least one parent who experienced a reduction in anxiety and depressive symptoms (assessed by a reduction at the HADS response by 1.6) during the intervention period from T0 to T2. The ICUR will be additional costs per improvement in parents’ quality-adjusted life years (QALYs) in a family, given as an equally weighted sum of QALYs of all parents, during the intervention period from T0 to T2. QALYs are based on the EQ-5D-5L [19] and evaluated by a German tariff [20] to generate utilities. The health economic evaluation will be conducted from the perspective of the SHI and the society.

The effect parameter and the EQ-5D-5L values are taken from the trial. Intervention costs (family-SCOUT salaries and travel costs) derive from the study documentation. Costs regarding physician contacts, hospital stays, medication intake, domestic help, inpatient care, and absent days are received from SHI data during the intervention phase (T0 to T2). In order to account for a societal perspective in the health economic evaluation, additional information is collected via questionnaires at T0 and T2, i.e., absent days as well as out-of-pocket costs induced at physician contacts, therapist contacts, domestic help, and rehabilitation. Indirect costs due to absent days will be evaluated by the human capital approach [21]. Moreover, we will consider family spillover effects as suggested for health economic evaluations with externalities [22, 23] by including health care costs of children which we receive also from SHI data. In particular, spillover effects are introduced based on the approach suggested by Al-Janabi et al. (2016) [24]. Costs and QALYs will not be discounted due to the short intervention period.

The statistical analysis of the health economic evaluation will follow the intention-to-treat approach. Multiple imputations to account for missing data will be conducted by considering the hierarchical structure of the data (regions and families). In sensitivity analysis, a full-case analysis will be conducted only with those families with complete data. The ICER and ICUR will be calculated based on the respective outcomes and costs of parents within the family (sum of costs induced by all parents of each family) whereas children’s costs are considered as a spillover effect. As health care use might be substantially higher previous to death and the intervention is not intended to have an effect on mortality, in sensitivity analysis, we will rerun the statistical analysis without families that experienced the death of a parent during the intervention phase. The non-parametric bootstrap method will be employed to generate 95% confidence intervals [25, 26]. In order to account for uncertainty, results will be presented on the cost-effectiveness plane and as a cost-effectiveness acceptability curve [27–30].

### Qualitative data analysis

Qualitative content analysis of the transcripts will be carried out [18]. Triangulation of the resulting codes and categories with the standardized questionnaire data will take place on the basis of a mixed-methods matrix [31]. This allows personal and family-related qualitative and quantitative characteristics to be linked in order to identify and contrast-specific typologies or patterns that can be attributed to the intervention.

### Study monitoring

The intervention and data collection will be accompanied by continuous study monitoring and a formative implementation evaluation. The monitoring activity will ensure that the standardized training of the family-SCOUTs at both intervention sites and the intervention is in accordance with the study manual and that documentation is uniform and complete. Any divergences from the manual will be documented and adopted to later implementation strategies and potential revisions of the manual. In addition, the consent process of family-SCOUT is also quality assured at all sites through monitoring activities; the same applies to the data transmission routes in the evaluators’ data management. Monitoring will comprise regular phone calls, semi-structured interviews, and audits. It will cover compliance with the study protocol, compliance with the manual, observance of the inclusion criteria, completeness of documentation, unexpected events, and patient dropouts. Due to the noninvasive and need-oriented nature of the intervention, no termination criteria will be initially defined. However, unexpected events and their potential consequences for the families or staff will be documented and discussed at regular project meetings.

In addition, the numbers of enrolled patients and other organizational data are monitored and sent weekly to the consortium leader (RWTH Aachen University) and evaluators (CHSR Bonn). The study progress is reported quarterly and discussed with the whole consortium according to an approved milestone plan; for the oversight of data management, a group consisting of members of the evaluators and consortium leader was also established. All events, progress data, milestones, and failures are reported quarterly to the funding agency.

## Discussion

Parental cancer can put a strain on the whole family and often disrupts daily routines. Families with underage children often experience organizational and emotional limits and fail to organize essential support for themselves. Available services are insufficiently frequented and many social services end with the death of the affected parent. Underestimating the impact on all family members and psycho-social and emotional strains can lead to severe health problems and an increased rate of psychological complications in the later course. This problem is addressed by family-SCOUT, whose innovative concept offers affected families and their children a novel kind of support. A family-SCOUT is a steady contact person who advises, accompanies, and supports families according to their needs. Together they develop individual solutions beyond the limits of sectoral care. Supporting a family in which one of the parents has developed cancer is a complex challenge. With its outreach, cross-sector, and cross-phase concept that offer organizational, emotional, and communicative support, family-SCOUT aims to reduce stress and prevent secondary mental illnesses.

For the reasons outlined above, the hospitals’ psycho-social services staff are not adequately prepared for such complex interventions, and interventions that start too early are often unsuccessful [2]. A characteristic of the presented intervention in contrast to existing services is the “active outreach approach” with the aim to minimize barriers to support access. It is expected that through family-SCOUT, families can overcome some of the challenges they face after a cancer diagnosis is given to one of the parents. The characteristics of family-SCOUT, especially the low-threshold access, reduce barriers for families to take up the service, increases a broad acceptance amongst families, and reach families who previously had no access to support services. Currently poorly developed inter-sectoral links between the different service providers involved often lead to uncoordinated and structurally insufficient care for the families. This phenomenon is further accentuated by the heterogeneity of care structures involved as well as the fact that they are mostly based on local initiatives with variable goals [8]. It is therefore necessary to evaluate and establish approaches for comprehensive, structured care for families with minor children and parental cancer approaches that also can be flexibly adapted to the individual needs of all family members. Therefore, the concept of family-SCOUT is designed to meet the specific needs of the families and offers an entire range of support; ranging from support regarding social and financial issues, to application support for domestic help, or promotion of disease management among parents and children. Most existing support services are severely restricted in time and manpower and consequently, their provided service is unstable, non-continuous, and often restricted to the treatment of the affected cancer patient within a respective institution. Consequently, psychological burdens on the children that develop and/or manifest themselves later in the course of the disease or years thereafter are neither registered nor treated [8]. The innovative intervention approach of family-SCOUT is capable of relieving existing structural deficiencies for affected families and providing timely, needs-adjusted access to adequate support in a structured way. It can be assumed that this will be able to reduce the number of psychological sequelae and the associated healthcare costs for all members of the family.

### Limitations and generalizability

The study design is non-randomized and non-blinded. This might cause a bias between IG and CG, which could affect the main effect of the primary outcome. Differences in distributions of baseline variables between IG and CG will be discussed carefully. Some families that meet the inclusion criteria will not be included due to missing informed consent. Reasons for initial non-response will be documented. There might represent a selection bias in terms of greater enrolment of families who have time and resources to participate, although the outreaching time-flexible concept tries to counteract this. As the primary research data is based on a self-completion questionnaire survey, there might be a selection bias in terms of higher educated families with sufficient German language skills or without migration history. Families from the CG might be more likely to drop out than families from the intervention group, as a greater benefit can be expected there. At the time of inclusion, the affected parents may be at different stages of the disease. Patients may have been diagnosed with cancer only recently, and others may be post-recurrence, while another group may be in a palliative situation. Estimated effects and costs in the subpopulation with SHI data might be biased from this selection. In addition, the study recruits participants at four locations within Germany, which are located in immediate proximity, limiting the external validity. Caution should be applied with respect to generalizing the results.

### Trial status

Recruitment of participants for the study commenced in October 2018, and the first family was included on October 9, 2018. On September 29, 2020, 406 families have been assigned, 216 into IG and 190 to CG; currently, 45 families have participated in the semi-structured interviews. The estimated enrollment period, including recruitment and inclusion, is anticipated to be 27 months (December 31, 2020) and will conclude, when the estimated sample size has been included. The version number and date of the protocol are v1.0 and July 6, 2018, respectively.

## Supplementary information

**Additional file 1:.** SPIRIT 2013 Checklist: Recommended items to address in a clinical trial protocol and related documents*

## Data Availability

The datasets generated and/or analyzed during the current study are not publicly available due to German laws on privacy protection but are available from the corresponding author on reasonable request.

## Abbreviations

BOÄ: Berufsordnung für Ärzte und Ärztinnen, Professional Code of Conduct for Physicians
CaPIN: Cancer Patient Information Needs
CG: Control group
CHSR: Center for Health Communication and Health Services Research
CIO: Center for Integrated Oncology
CONSORT: Consolidated Standards of Reporting Trials
COSIP: Children of Somatically Ill Parents
DT: Distress thermometer
EQ-5D: EuroQoL-5D (questionnaire)
FAD: Family Assessment Device
FLZ: Fragen zur Lebenszufriedenheit-Module, Questions on Life Satisfaction Module
F-SCOUT: Cross-sector and cross-phase support for families with parental cancer
HADS: Hospital Anxiety and Depression Scale
HLS-EU-Q16: Health Literacy Survey, European Union, short version
HRQoL: Health-related quality of life
ICD-10: International Statistical Classification of Diseases and Health-Related Problems
ICER: Incremental cost-effectiveness ratio
ICUR: Incremental cost-utility ratio
IG: Intervention group
MID: Minimal important difference
MOS-SS: Medical Outcomes Study Social Support Survey
MRC: Medical Research Council
NCCN: National Comprehensive Cancer Network
PA-F-P-KF: Fear of Progression Questionnaire, Short Form
PCQ: Parenting Concerns Questionnaire
SHI: Statutory health insurance
QALY: Quality-adjusted life year
